# Artificial CT images can enhance variation of case images in diagnostic radiology skills training

**DOI:** 10.1186/s13244-023-01508-4

**Published:** 2023-11-07

**Authors:** Elfi Inez Saïda Hofmeijer, Sheng-Chih Wu, Rozemarijn Vliegenthart, Cornelis Herman Slump, Ferdi van der Heijden, Can Ozan Tan

**Affiliations:** 1 Robotics and Mechatronics, Faculty of Electrical Engineering, Mathematics and Computer Science, University of Twente, Enschede, The Netherlands; 2 Dept of Radiology, University Medical Center Groningen, University of Groningen, Groningen, The Netherlands; 3 Data Science Center in Health (DASH), University Medical Center Groningen, University of Groningen, Groningen, The Netherlands

**Keywords:** Artificial image, Artificial intelligence, Radiology, Medical image education, Personalized education

## Abstract

**Objectives:**

We sought to investigate if artificial medical images can blend with original ones and whether they adhere to the variable anatomical constraints provided.

**Methods:**

Artificial images were generated with a generative model trained on publicly available standard and low-dose chest CT images (805 scans; 39,803 2D images), of which 17% contained evidence of pathological formations (lung nodules). The test set (90 scans; 5121 2D images) was used to assess if artificial images (512 × 512 primary and control image sets) blended in with original images, using both quantitative metrics and expert opinion. We further assessed if pathology characteristics in the artificial images can be manipulated.

**Results:**

Primary and control artificial images attained an average objective similarity of 0.78 ± 0.04 (ranging from 0 [entirely dissimilar] to 1[identical]) and 0.76 ± 0.06, respectively. Five radiologists with experience in chest and thoracic imaging provided a subjective measure of image quality; they rated artificial images as 3.13 ± 0.46 (range of 1 [unrealistic] to 4 [almost indistinguishable to the original image]), close to their rating of the original images (3.73 ± 0.31). Radiologists clearly distinguished images in the control sets (2.32 ± 0.48 and 1.07 ± 0.19). In almost a quarter of the scenarios, they were not able to distinguish primary artificial images from the original ones.

**Conclusion:**

Artificial images can be generated in a way such that they blend in with original images and adhere to anatomical constraints, which can be manipulated to augment the variability of cases.

**Critical relevance statement:**

Artificial medical images can be used to enhance the availability and variety of medical training images by creating new but comparable images that can blend in with original images.

**Key points:**

• Artificial images, similar to original ones, can be created using generative networks.

• Pathological features of artificial images can be adjusted through guiding the network.

• Artificial images proved viable to augment the depth and broadening of diagnostic training.

**Graphical Abstract:**

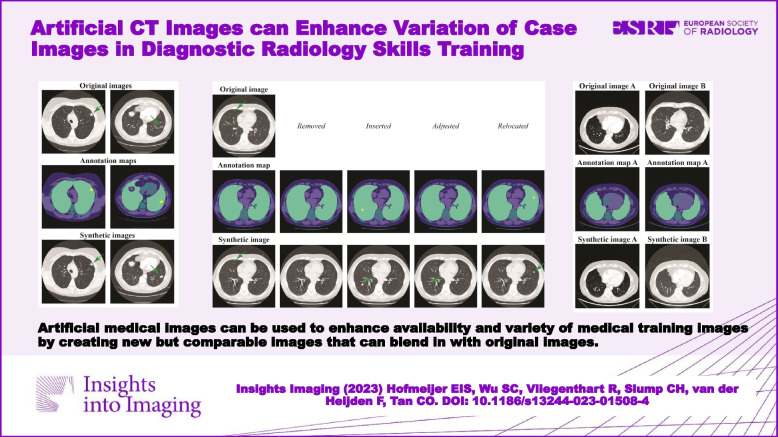

**Supplementary Information:**

The online version contains supplementary material available at 10.1186/s13244-023-01508-4.

## Introduction

Diagnostic radiology training requires wide availability of variable but representative images whose features are accurately delineated and/or annotated. In fact, the quality of diagnostic skills positively correlates with the volume of, and thus, variability among practice cases [1]. Furthermore, the trainee benefits from interactive, case-based learning, where the cases include an ample variety and are suited to trainee’s level of experience [2, 3]. To this end, simulated training in diagnostic radiology has emerged as a means to augment the depth and broadening of available training cases. The advancement of artificial intelligence (AI) techniques all but enforced the idea of tailoring training options for trainees’ level and performance, or “precision education” [3–5]. Underlying this idea is the observation that training should promote trainee’s experience with a variety of cases but in accordance to trainee’s diagnostic skills, without increasing the workload of the educator.

Simulated training can rely on existing material and/or “new” artificial (or so-called “synthetic”) ones could be created. Most applications promote active learning using existing medical images, [6] and some allow educators to assign cases to individual trainees to promote their exposure to variable and interesting cases [7]. Such applications do enable precision education and could offer extra learning opportunities without the requirement of additional workload for educators. However, they still require a sufficiently large number of cases, which are often difficult to secure. AI-based synthetic images have been suggested to aid training in different contexts [8–10].

The representation of synthetic images can be created while adhering to specific conditions and constraints. In other words, synthetic images can be created in real time to conform the experience or variety of cases a trainee will see. However, to be useful in radiology education, it is critical that the created synthetic images “look” sufficiently similar to the original ones and that they enable precise control over image characteristics.

We sought to investigate if semantic image synthesis networks can create images that can sufficiently blend in with real radiographic images and whether they allow a reasonable degree of control of anatomical and pathological features.

## Methods

### Data

As a use-case for this study, we used the publicly available Lung Image Database Consortium and Image Database Resource Initiative (LIDC-IDRI) data set [11–13]. While radiographic lung images are readily available at sufficient volume for training, our choice was deliberate. First, this data set contains a large number of clinical CT scans: 1018 diagnostic and screening scans, both with and without pathological features (lung nodules). Second, the images were obtained using either standard or low-dose CT. Third, relevant lesions and features were annotated by four experienced thoracic radiologists via consensus, though images were not annotated with lesion attributes. LIDC-IDRI comprises CT scans from different institutions and individuals, making it a diverse dataset in terms of patient demographics. However, specific demographic information about the patients, such as age, sex, and ethnicity, are not available publicly due to privacy concerns [13]. Thus, this data set provided a unique opportunity to test the reliability, realism, and utility of synthetic images for diagnostic radiology training; our methods (and results; below) are agnostic to the image modality, body part, or pathological features.

Upon retrieval, slices in each scan were harmonized by clipping the Hounsfield Units at [−1350, 150], the lung window [14]. 2D slices were retained at their original resolution of 512 × 512 pixels, and 17% of all 2D slices contained lung nodules. Detailed information about scan selection and pre-processing of 2D images can be found in Appendix 1 section A1.

### Semantic image synthesis

**Fig. 1:**
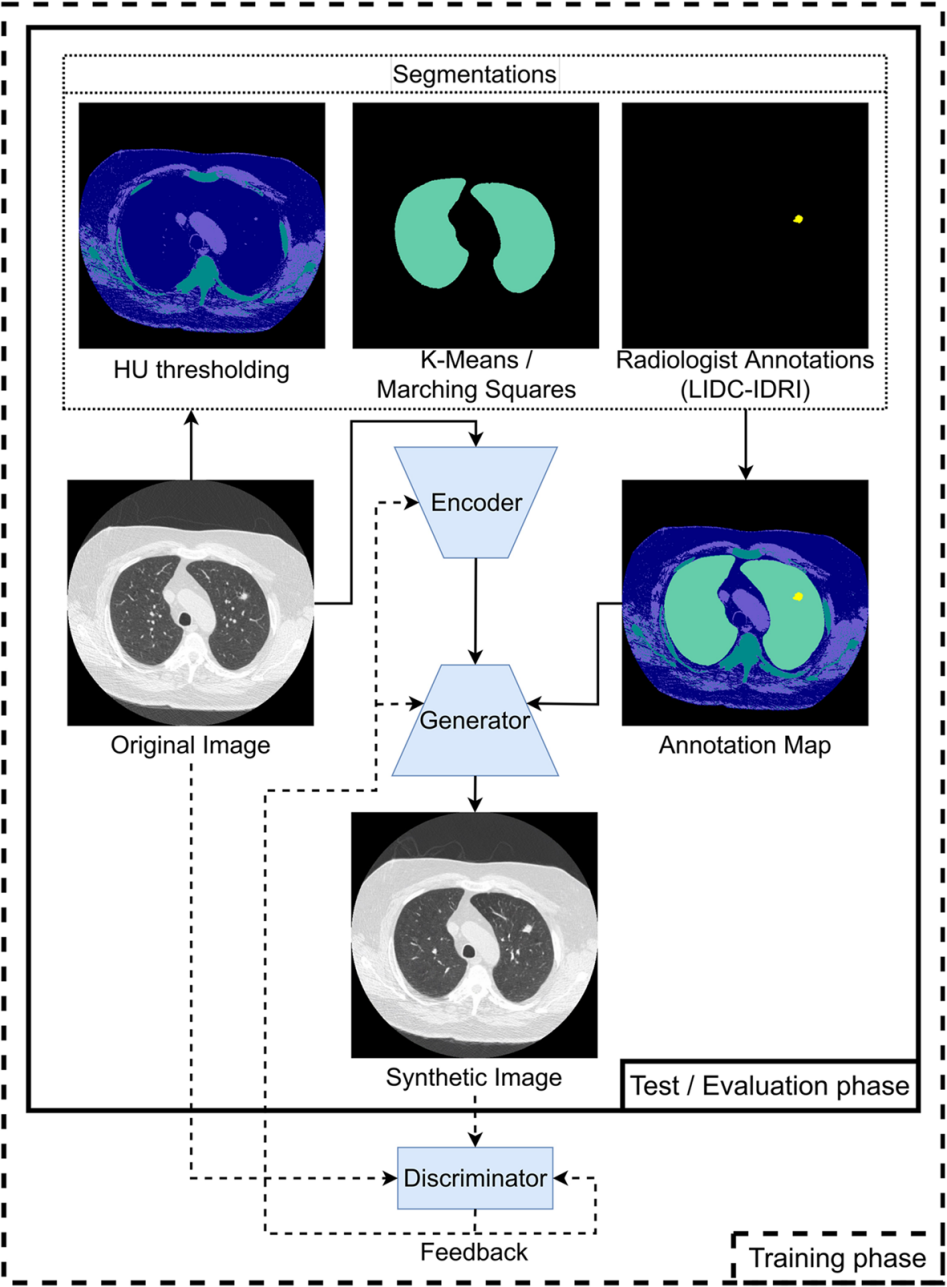
Workflow of obtaining the annotation maps and training and testing the semantic image synthesis network. The semantic image synthesis network consists of three models (light blue): the encoder, the generator, and the discriminator. It takes an annotation map as input and is *additionally* guided by an original image. The feedback from the discriminator allows all three models to learn during training and is discarded during evaluation

Semantic image synthesis networks, a type of AI, provide a unique opportunity to realize precision education and to improve diagnostic skills. These networks constitute a specific type of generative adversarial network. They rely on additional “semantic” information to create (“synthesize”) the gross image features. This information is embedded in an image or map through labeling of, for instance, separate organs or pathological features. This way information can be introduced to the synthetic image that was different (or even not present) in the original, actual image (see Fig. 1).

In this work, we used the network developed by Park et al. [15], which seeks to better preserve the semantic information in the synthetic image. In this context, semantic information is described as the information about which pixel belongs to which object or group in an image. We chose this network mostly because of its potential to create a variety of images and partly because of the ease of implementation, not necessarily to create the “most realistic” synthetic image. The pipeline for training and evaluation of the network is depicted in Fig. 1. Further details of the training and network parameters are described in Appendix 1 section A1. All code necessary to replicate our results is provided at https://github.com/UT-RAM-AIM/Realism-Study.

### Annotation maps

The semantic image synthesis network utilizes a map of pre-determined features to create images similar to, but distinct from the original images. This allows them to be guided to manipulate arbitrary features (a.k.a. “ annotation maps”; in our case, anatomical and pathological characteristics) of the output synthetic image. In this work, we used annotation maps akin to a segmentation map, that reflect each of the major objects in the images. It is the same size as the 2D image slices, 512 × 512 pixels, and can offer guidance and constraints to the shape and location of the target features.

The annotation maps in this work included five labels. We algorithmically segmented the original LIDC-IDRI images to obtain annotations that identify the full body, soft tissue, dense tissue, and total lung area (Appendix 1 section A1). Manual annotation provided by LIDC-IDRI was used to delineate lung nodules, if any, as the fifth label. These five labels were deemed to contain enough information, based on known anatomy, to guide the semantic synthesis network.

Original images and corresponding annotation maps were randomly split into a training and validation set and an independent test set. Details about obtaining the annotation map, data split, and selection of the slices are described in Appendix 1 section A1.

### Quality evaluation

To assess whether synthetic images (primary set) can be used along with the original ones, we created additional synthetic images of lesser quality than the primary set. Specifically, as negative controls, we created a set of synthetic images that are of reasonable quality, but have serious flaws (control set 1), and another set that was obviously not real (control set 2). This was critical to evaluate the extent to which the primary synthetic image set can blend in with the original image set, relative to those with deliberately low realism and those that are obviously unrealistic. To this end, we trained the network for a second and third time with, respectively, 2% and 0.3% of the main training data set. Details about data split and network training for both subsets can be found in Appendix 1 section A1. This way, we ensured that the apparent quality of the primary set was not due to other intrinsic factors (e.g., attention paid by the radiologists, their experience, etc.).

The degree to which the synthetic images are similar to original images can be assessed both quantitatively and qualitatively. However, for quantitative metrics, there is no consensus on a single best metric and their validity in clinical setting [16, 17]. In particular, quantitative metrics may not always reflect expert judgment [18–21]. Though qualitative, perception of domain experts is a valid, reliable, and interpretable approach that also signifies clinical relevance of the synthetic images [8, 19, 20, 22]. To determine if these four sets ((1) original images, (2) primary synthetic set, (3) synthetic control set 1, and (4) synthetic control set 2) are distinguishable, the sets of images were evaluated both quantitatively and qualitatively.

As the main quantitative metric, we used the Structural Similarity Index Measure (SSIM). The SSIM is based on pairwise comparisons, i.e., comparison of the original image with the corresponding synthetic image. It ranges from 1 to 0 representing, respectively, identical and completely dissimilar images. This choice was based on the consideration of interpretability of the metric: since multiple types of metrics are available that assess different properties of the synthetic image compared to the original one, we also derived four other common metrics; see Appendix 1 section A2. The impact of the size of the training set in the primary synthetic set and the two control sets was tested for statistical significance using a one-way ANOVA followed by a post-hoc Tukey’s test. We tested the hypothesis that the primary synthetic set will achieve a SSIM score closer to 1 compared to control sets 1 and 2.

In addition to quantitative measures, we sought expert opinion based on previous approaches [19]. Five radiologists were asked to assess 60 quartets of 512 × 512 images. Three out of the five were board-certified radiologists in the Netherlands and one was board-certified in the USA, all with > 10 years of experience in thoracic CT. The fifth was a radiology fellow in The Netherlands with 3 years of experience in thoracic CT. Every quartet contained one original image, selected randomly from the test data set, and three synthetic images (primary set, control set 1, control set 2). All synthetic images were generated using the same original annotation map that corresponds to the original image. The radiologists were presented with a quartet, with the location of each image within the quartet assigned randomly. The radiologists were blinded to which image was the original one. First, the radiologists were asked to indicate the image that is the original image. Second, the radiologists were asked to score the quality of each image in a given quartet on a scale from 1 (unrealistic) to 4 (almost indistinguishable from the original image). Ordinal regression was used to test the ranking of the expert rating across original and synthetic images created. We tested the hypothesis that radiologists are able to distinguish original and synthetic images.

### Manipulating annotations

To explore the capabilities of the semantic image synthesis network, we manipulated information in the annotation map that corresponds to the main pathology, lung nodules, as it is the most clinically salient feature. In particular, we used removal, insertion, and relocation of the lung nodule label in the annotation map. Through this approach, we investigated if the resulting synthetic images also adhere to these new constraints.

A second option to provide guiding information is to provide an original image to the network. In this case, the new synthetic image will have the appearance of that particular guiding original image. This adjusted synthetic image will still adhere to the annotation map and therefore only reflect variability due to, e.g., scanner differences such as visibility of a gantry or overall intensity in the image. We also explored this “example-guided synthesis” by guiding the network with different original images, while keeping the annotation map the same.

## Results

**Fig. 2:**
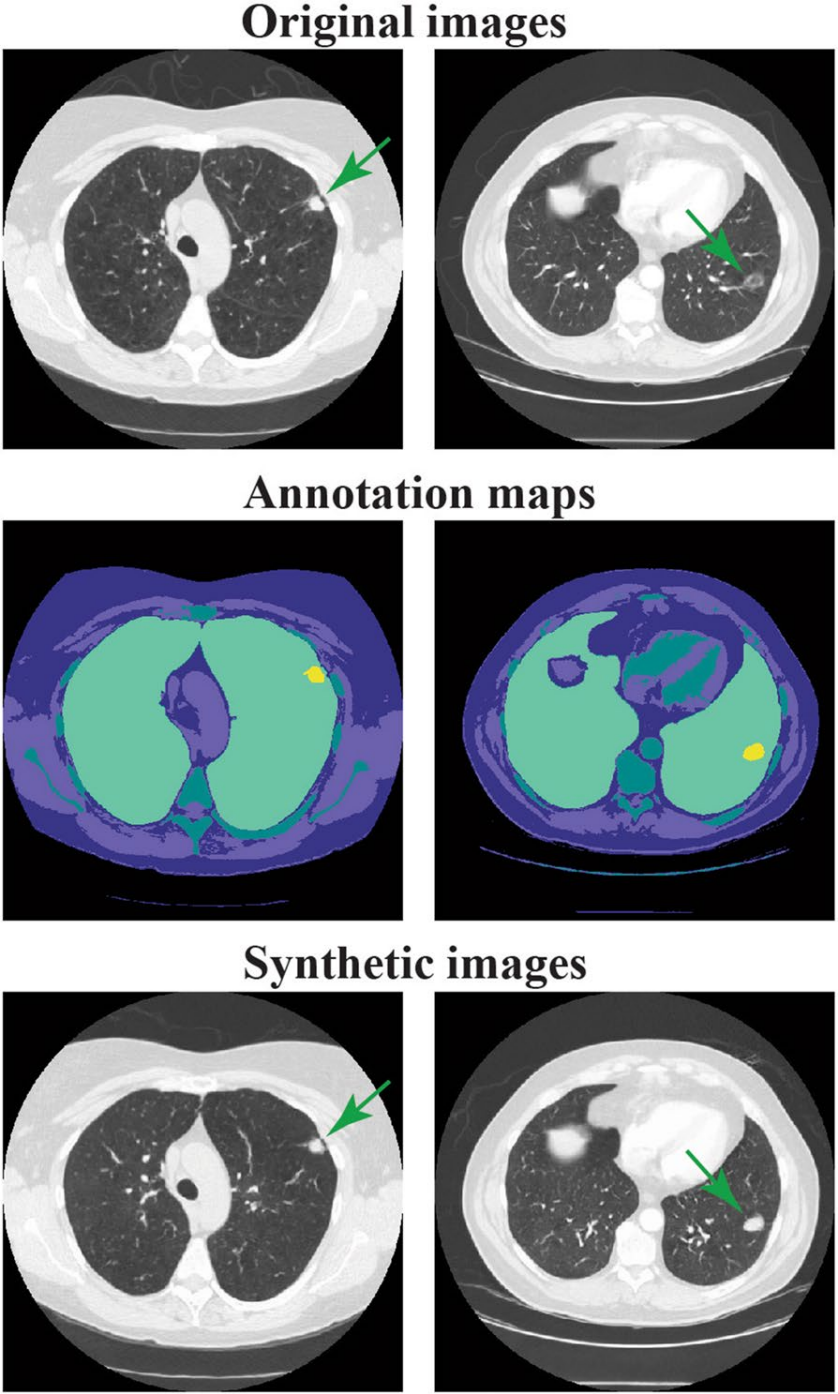
Two examples of original images (top) with corresponding annotation maps (middle) and synthetic images (bottom) from the trained semantic synthesis network. The colors of semantic label maps indicate the five labels; body, soft tissue, dense tissue, lung area, and nodule area

**Fig. 3:**
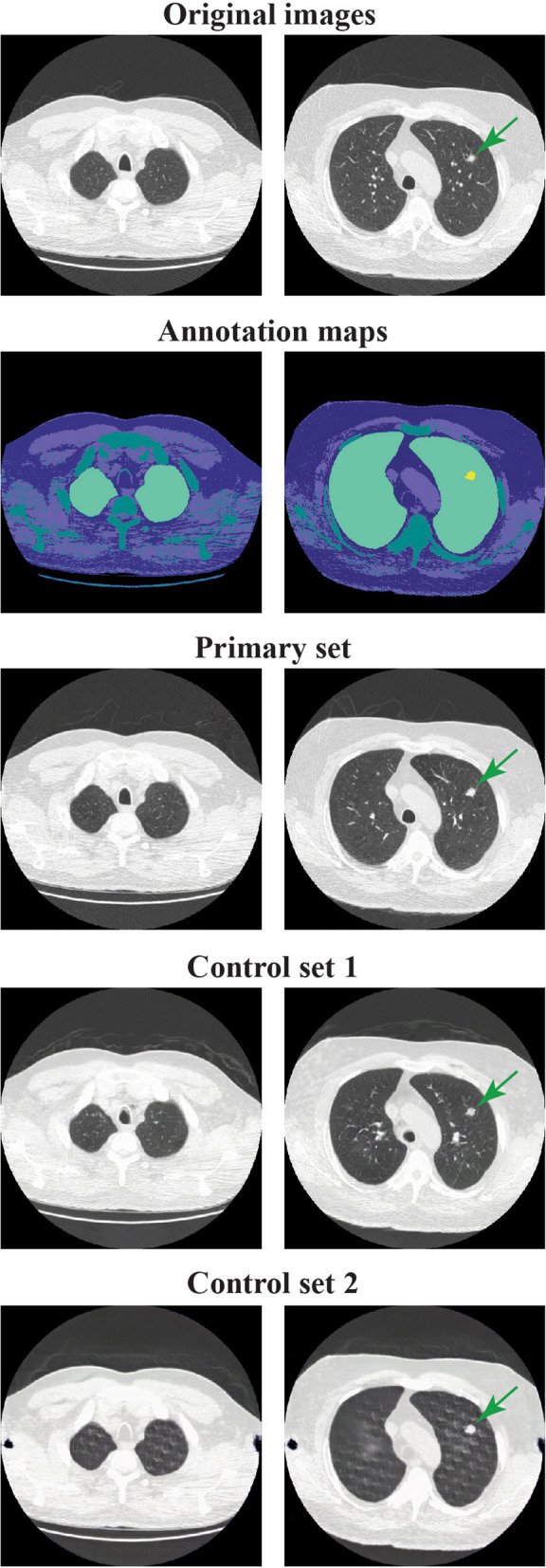
Two examples of original images (row 1) with corresponding annotation maps (row 2) and resulting synthetic images (rows 3, 4, and 5) from the trained semantic synthesis network with 100% (primary set), 2% (control set 1), and 0.3% (control set 2) of the data, respectively

After pre-processing, the final training data set consisted of 39,803 slices from 806 individuals (see Appendix A1 for scan and slice selection). Trained synthesis network was evaluated using a test set of 5121 slices from 90 scans, resulting in a created synthetic set of also 5121 images. Visual inspection confirmed that large anatomical structures in the synthetic images corresponded to those in the original image (Fig. 2). This was done to ensure that synthetic images adhered to the constraints of anatomical structures indicated by the annotation map. In addition, we inspected synthetic images to ensure the absence of artefactual structures that were not guided or constraint by the annotation map, inside the lung, and/or nodule area. Inside the lung area differences can be seen in the visualized pulmonary vessels. Furthermore, the lung nodule appears more solid in the synthetic image. Synthetic images of the primary set show more detail than those from control sets 1 and 2 (Fig. 3).

Synthetic images generated from the primary set attained an SSIM of 0.78 ± 0.04 (95% CI [0.74, 0.75]). Those generated from control sets 1 and 2 attained an SSIM of, respectively, 0.78 ± 0.05 and 0.76 ± 0.06. The mean SSIM of the three distributions, the primary set and two control sets, was significantly different (*F* = 175.67, *p < *0.001). Tukey’s post-hoc test showed that this difference was driven by the network trained on control set 2 (*p* = 0.98 for primary set vs control set 1, *p < *0.001 for primary set vs control set 2, and *p < *0.001 for control set 1 vs 2). The results of the additional metrics are shown in Additional file 1: Appendix A2.

To obtain expert opinion, 60 quartets of images were assessed by four radiologists. A fifth radiologist completed assessment of 21 quartets. Therefore, in total, 261 quartets were evaluated. In 93.1% and 100% of the quartets the synthetic images from, respectively, control set 1 and control set 2 were correctly identified as not original (Table 1). In contrast, in over a fifth of the quartets (21.5%), our synthetic images created using the primary set were identified as the original image. Of the first 21 quartets, 3 were identified as the original image by all five radiologists, 7 by four, and 11 by three or less. Of the remaining 39 quartets, 18 were identified correctly by all four radiologists, 11 by three, and 10 by two or less.

**Table 1:** Domain expert scoring of original and synthetic images. The second column shows the percentage of each set identified as the original image. The third column shows the average score radiologists have assigned to images in each set

Data set	% identified as the original image	Average score
Original images	71.7%	3.73 ± 0.31
Primary set (39,803 images)	21.5%	3.13 ± 0.46
2% subset (control set 1)	6.8%	2.32 ± 0.48
0.3% subset (control set 2)	0%	1.07 ± 0.19

Primary synthetic images achieved the highest score after the original images, with an average score (1–4) of 3.13 ± 0.46, somewhat lower than the original images (3.73 ± 0.31). As expected, synthetic images from the two control sets scored lower, with ratings of 2.32 ± 0.48 and 1.07 ± 0.19. The mean expert score was different for at least one of the distributions (*F* = 987.68, *p < *0.001). Tukey’s post-hoc test showed pairwise differences across images from all sets (*p < *0.001). Consistent with these results, radiologists’ score was closely related to the set (primary, control set 1, control set 2) a synthetic image originated from (ordinal regression, *p < *0.001).

### Manipulating annotations

**Fig. 4:**
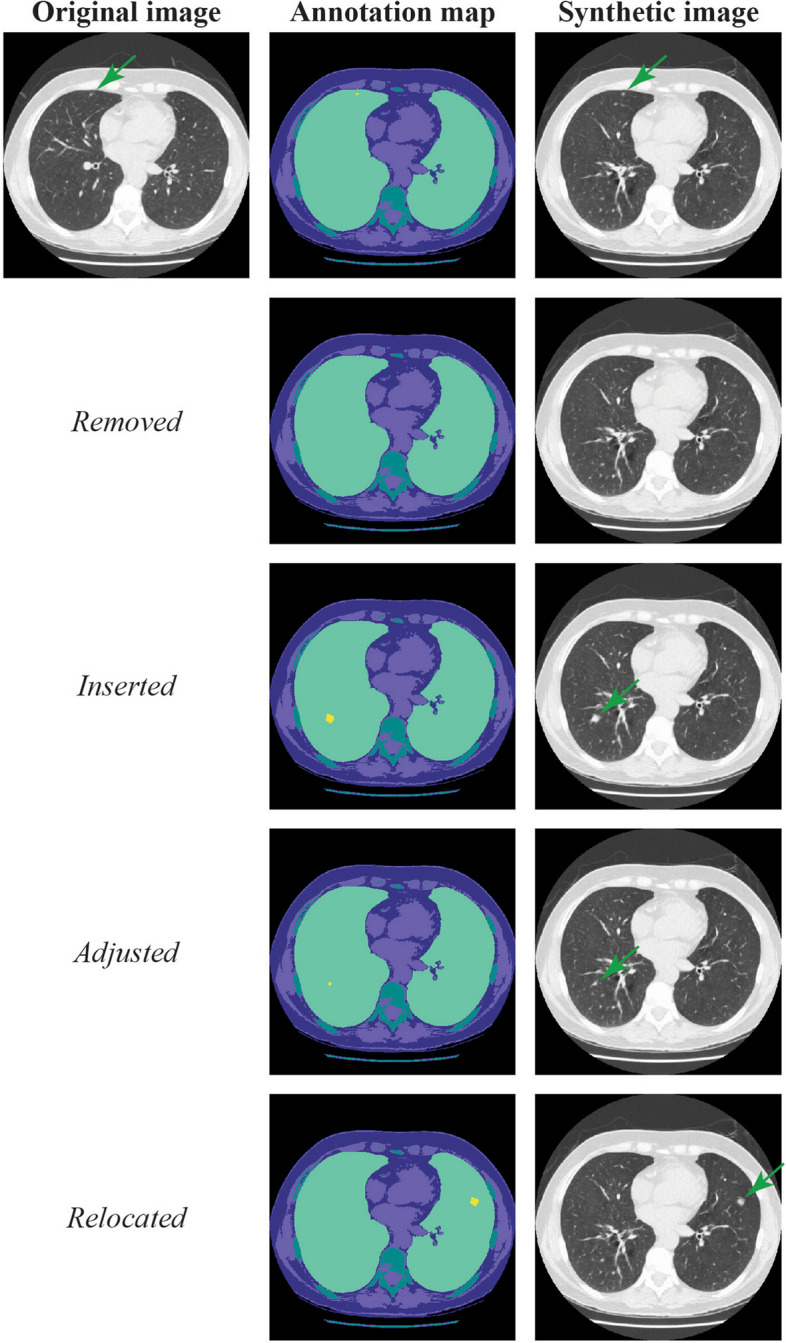
Examples of manipulation of the lung nodule label in the annotation map and corresponding synthetic image. The first row shows the original image with corresponding annotation map and synthetic image. In the second row, the original nodule label is removed. In the third a different one is added in a different position. Rows four and five show adjusting and relocating the nodule label respectively. Dark green arrows indicate the (synthetic) nodule

**Fig. 5:**
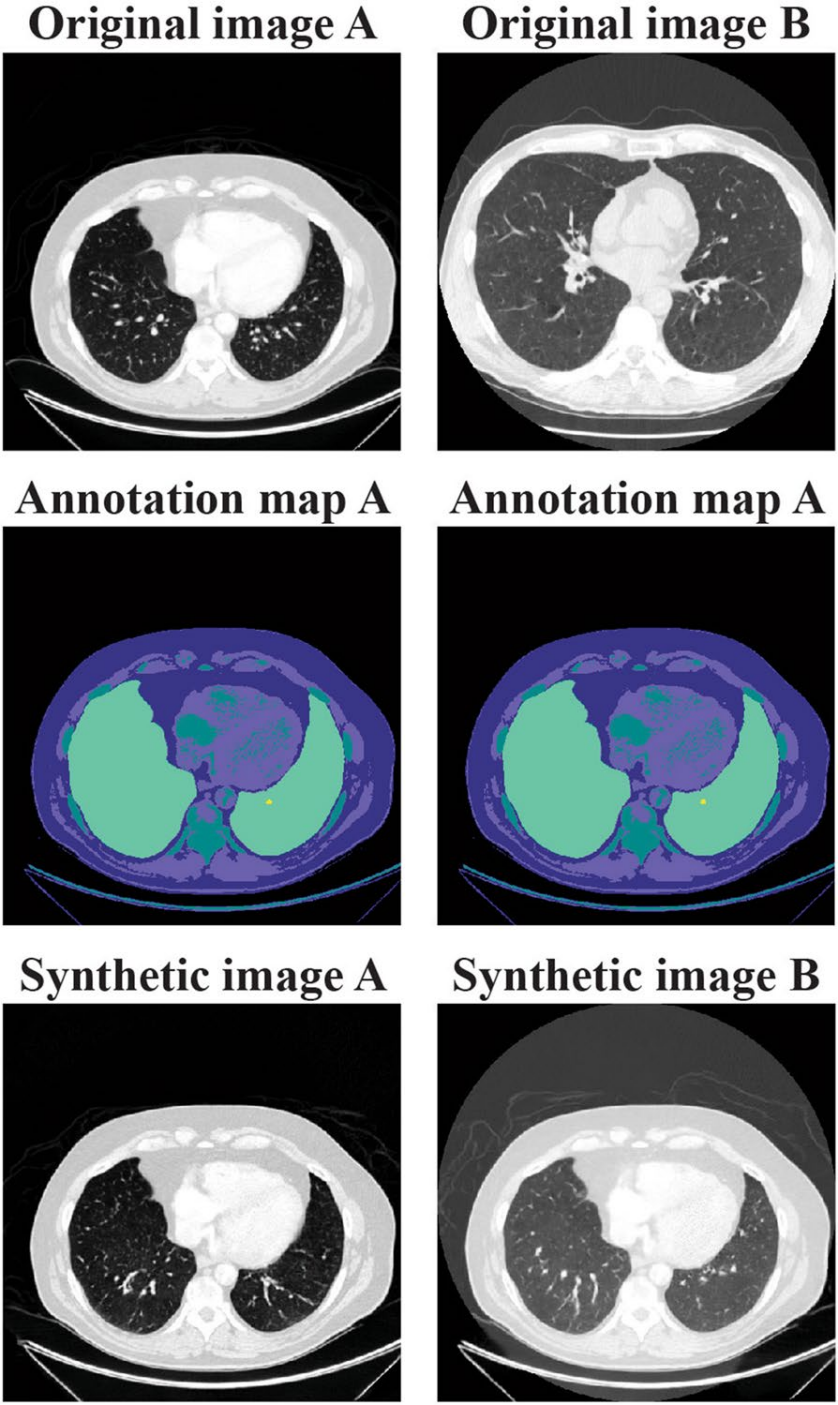
Example-guidance capabilities of the semantic synthesis network. The top row shows the original images used to additionally guide the network, followed by the (same) annotation map in the second row. Using different guiding images results in a synthetic image with a different appearance and intensity (bottom row)

After removal, insertion, adjustment, and relocation of the primary pathology (in our case, lung nodules), synthetic images adhered to these constraints provided by the annotation maps (Fig. 4). Furthermore, the network was additionally guided by two different images obtained using a different scanner with variable visibility of the gantry and overall intensity. The appearance of the synthetic images did change in parallel to the guidance scanner view, but still adhered to the constraints imposed by the annotation map (Fig. 5).

## Discussion

Our results show that synthetic images can be created as additional “new” images similar to the original ones and that their pathological features can be varied through manipulation of the guiding annotation map. This demonstrates the feasibility of medical image generation with full field-of-view, with and without pathological features, and without the need for explicit manual annotation. Synthetic images can be manipulated to add to the breadth of the cases seen by the trainee in an adaptive way tailored to their level of experience in real time.

As expected, independent radiologists were able to identify the original image for most cases. However, in over 20% of the cases, they did identify synthetic images as “original.” This supports our initial hypothesis that synthetic images are reasonably realistic. In fact, while the radiologists were able to successfully identify unrealistic images (negative control sets 1 (93%) and 2 (100%)), their average rating of synthetic images was close (3.13) to what is expected from an original image. This was higher than the rating of negative (i.e., deliberately unrealistic) controls. There may be characteristics of the synthetic images that influence radiologists’ ability to distinguish original and synthetic images. However, these can be reader-dependent and cannot always be explicitly defined (e.g., “vascular structures are too vague”). It could be interesting to assess this in future research as it can point toward potential improvements for image synthesis.

It is important to note that a number of similar prior studies relied on a binary rating (real or not real) [23–25]. This is in contrast to our approach, where radiologists were asked to identify the original images among four synthetic ones, and to rate the quality of both synthetic *and* original images. This design is deliberate; it has the advantage of probing the ability of radiologists to discriminate between original and synthetic images (as opposed to “guessing” the latter). Therefore, it allows us to not only probe the quality of the images, but also to objectively quantify their degree of quality compared to a negative control. We cannot assess to what extent a synthetic image can “pass the realism test” in the absence of the corresponding original image available for viewing alongside the synthetic one. Nonetheless, our results do demonstrate that synthetic images may blend in with original ones, augmenting the pool of images to facilitate the training of radiologists.

Quantitative similarity metric results were less expected. In particular, while synthetic images attained an SSIM of 0.78 on average, this was not significantly different from the SSIM attained by control set 1 (0.78). It is difficult to compare the obtained SSIM to results from other studies, since synthetic images are created at different resolutions [26] or with some regions masked out to focus on areas of interest [27]. Therefore, there is no established benchmark. Scores vary from 0.25 [28], to 0.80 [29] and 0.89 [27] in other studies similar to ours.

In our study, SSIM scores were in contrast to our expectation that the primary synthetic image set would have a higher mean similarity than control set 1. This is likely to be related to the apparent validity of these quantitative metrics (or the lack thereof) [21]. SSIM is primarily a metric of the similarity vis-a-vis structure, contrast, and luminance. Therefore, it is likely that SSIM reflects the similarity of images in structural appearance in contrast to the similarity in terms of perception. We cannot reconcile their apparent mismatch with radiologist’ scores, nor can we ascertain whether or how quantitative metrics are congruent with expert opinion. However, it is likely that the features a radiologist implicitly considers are more complex, at the interaction of structural, perceptual, and conceptual processing [30, 31]. Nonetheless, as we pointed out in the introduction, we relied primarily on expert opinion given their ecological validity and clinical relevance.

Our work also shows that features of synthetic images can be altered. This is similar to a prior study [32], which corroborates our findings that synthetic images are structurally editable through adjustment of annotation maps. However, the focus of the prior study was limited to the gross appearance of pathologic features (as a consequence of COVID-19 infection), whereas our work provides a more targeted and variable approach. Our study, along with the prior one, highlights the need for research on synthetic image generation with different organs and imaging modalities.

Our research is not without limitations. First, although we were successful in controlling the presence and location of the pathological feature (lung nodule) in the synthetic image, we did encounter a few artefactual features. For example, the texture or appearance of a nodule in a synthetic image, as was shown in Fig. 2, could be quite different compared to the original image. Furthermore, we observed that the texture was very similar across most synthetic nodules (see Appendix 1 Figure A1 for an example). This is likely a consequence of the amount of information about the nodules provided to the network during training, compared to other, much larger, structures in the images (e.g., lungs, bone structures). Therefore, during generation, the network may have primarily relied on an “averaged” texture of a nodule. Future work should explore how more detailed structural information can be incorporated during image generation.

Second, random assignment of the (synthetic) images presented to expert radiologists may have resulted in unintended consequences of occasional images that reflect the very last section of a scan, with minimal lung area. This may have facilitated experts’ recognition of synthetic images. This reflects a broader limitation of synthetic image generation. The quality of the synthetic images partly depends on the quality of the guiding annotation maps (“garbage-in-garbage-out”). Since the algorithm used to create the annotation maps is based on image segmentation, it will inherit all the inaccuracies present in the original image.

Lastly, the use-case in this work is focused on creating synthetic 2-dimensional images, which is in contrast to how radiologists review scans in the clinical setting. This limitation, in our case, was unavoidable. Creating 3-dimensional images require substantially higher computational power, beyond what was available to us. Furthermore, generating 3-dimensional images poses the additional challenge of annotating other features, such as vascularization in the guidance image. The LIDC/IDRI database does not provide information or annotations beyond lung nodules. However, to provide an experience as realistic as possible in precision education, future work should focus on 3-dimensional synthetic images.

Despite its limitations, our work highlights the feasibility and utility of synthetic images in the context of precision education in diagnostic radiology skills training. In particular, our ability to manipulate pathological features suggests that it may be possible to create synthetic images that reflect the presentation of different features, such as benign and malignant tissue. This, in turn, can open up new venues for exploring subtle alterations in tissue characteristics that impact pathological presentation, to explore cross-modality translation of medical images, or to facilitate implementation of learning programs where each individual (or algorithm) is presented by a series of (synthetic) images that are tailored to existing skill level. Moreover, although our use-case was chest CT with lung nodules, our results support the feasibility and potential utility of similar approaches for other imaging modalities, organs, and/or pathologies. While our study was not designed to explore these applications, it provides an early step toward using synthetic images in this medical educational context.

### Supplementary Information

**Additional file 1: Appendix A1.** Appendix A2. Supplementary Figure 1. Supplementary Table 1. Supplementary Table 2. 

## Data Availability

The dataset used, Lung Image Database Consortium (LIDC) and Image Database Resource initiative (IDRI), is publicly available at The Cancer Imaging Archive (TCIA). All code for pre-processing, evaluation, and details about the used software libraries, packages, and model can be found at: https://github.com/UT-RAM-AIM/Realism-Study.

## Abbreviations

LIDC-IDRI: Lung Image Database Consortium and Image Database Resource Initiative
SSIM: Structural Similarity Index Measure
