# Supplementary material for: Artificial CT images can enhance variation of case images in diagnostic radiology skills training
Source: Insights Imaging. 2023 Nov 7;14:186. doi: 10.1186/s13244-023-01508-4 (PMC10630276; doi:10.1186/s13244-023-01508-4)
Supplement: Supplementary file 1 — Additional file 1: Appendix A1. Appendix A2. Supplementary Figure 1. Supplementary Table 1. Supplementary Table 2. [file 13244_2023_1508_MOESM1_ESM.docx]

Artificial CT Images can Enhance Variation of Case Images in Diagnostic Radiology Skills Training

ELECTRONIC SUPPLEMENTARY MATERIAL

Appendix A1

Pre-processing of Original Dataset

Before the semantic synthesis network was trained, the scans from LIDC-IDRI were pre-processed. The intensity values of the DICOM format scans were rescaled using the rescale slope and intercept supplied with each scan. Subsequently, the HU values were clipped to [-1350, 150], the lung window, and each 2D slice was resampled to a 1x1mm resolution ensuring the same range in pixel values. To ensure equal-sized slices, each slice was cropped or padded to obtain an 512x512 pixel image. Scans were excluded if the slice thickness was greater than 2.5mm. The individual 2D images were save in JPEG format with each pixel linearly scaled to [0, 255] and with a bit depth of 24. Subsequently, complementary guiding annotation maps were obtained for each 2D image.

Obtaining Annotation Maps

The first three labels of the annotation map are assigned according to the HU values in the slice: ’body’ between [-400, 150], ’soft tissue’ between [5, 145], and ’dense tissue’ between [145, 150]. These labels are obtained in this respective order and if there is overlap, the latter label will overwrite the former. The fourth label, ’lung area’, is obtained by applying a k-means classification and the marching squares algorithm [1,2]. Using two morphological operations, erosion and dilation, k-means classification was used to classify non-lung and lung pixels. The background was excluded by setting a maximum area threshold. However, this alone could not render a precise boundary and therefore the marching squares algorithm was additionally applied to obtain lung contours. These contours were transferred into masks and compared to the masks obtained by k-means using cosine similarity, realizing more exact lung contours. In some cases, this approach failed, and the resulting maps were manually adjusted to obtain a realistic lung mask. The ‘lung area’ label overwrites the former labels. The fifth label obtained covers ’nodule area’ and was determined by using the radiologists’ annotations from LIDC-IDRI and applying a 50% consensus criterion [3]. Nodule annotations were only taken into account if at least 3 out of the 4 radiologists had marked the nodule. This label overwrites the former labels. Lastly, the annotation maps were saved as 512x512 PNG images.

Data split and Balancing

In total 44,924 image – annotation map pairs were collected from 895 individuals. The 44,924 images were split patient wise into a training set of 806 individuals consisting of 39,803 image – annotation map pairs and a test set of 90 individuals consisting of 5,121 image – annotation map pairs respectively. In the test set were 29 scans of a scanner that was not present in the two Control Sets. Therefore we removed these scans from the test set to avoid biased results due to scanner differences. This reduced the test set to 61 individuals consisting of 3,488 image – annotation map pairs.

Nodules in this data set are mostly small and only occur in a small percentage of all the slices. There is therefore an imbalance in the amount of information that is given to the network about this ‘nodule area’ label compared to the other labels. To even this out, not every slice without nodules is used. From every patient case, 20% of all slices of each scan were used, including all nodule slices if any. To get a representation of the whole CT scan, the non-nodule slices were sampled uniformly throughout the scan. An exception to this can be found in the ~0.3% subset of the training set, since this set only contains 1 scan, this scan is used fully and therefore the 20% sampling rule is not applied. Table A1 can be consulted for more information about each set; scanner origin, the number of scans and corresponding 2D images, and number of nodules present.

Network training and parameters

The semantic image synthesis network consisted of three networks, taking an annotation map as the main input guidance and is additionally guided by an original image (Figure 1). The three networks are called 1) the encoder, tasked with encoding the appearance of the original image, 2) the generator, tasked with the synthesis of the synthetic image, and 3) the discriminator, tasked with assessing if its input is a synthetic or an original image. Together, the synthesis network is trained to generate a realistic image -- *synthetic image* -- based on the annotation map. This map offers guidance and constraints on shapes and their location to the synthesis network, while the original image guides the appearance (e.g., texture and intensity) of the synthetic image. The feedback from the discriminator allows all three networks to learn during training and is discarded during evaluation, since at that point we are only interested in the synthetic image that is produced by the generator.

The synthesis network was trained for 50 epochs with a batch size of 8. Existing code from Park et al. [4] is used and can be found at: https://github.com/NVlabs/SPADE. The learning rates for the generator and discriminator were set to 0.0001 and 0.0004 respectively. The Adam optimizer [5] was applied with β₁ = 0 and β₂ = 0.9. Also, the two time-scale update rule [6] was implemented and the learning rate was linearly decayed to 0 from epoch 25 towards epoch 50. The annotation maps were used as input and the corresponding original image was used to initialize the random vector input. Two NVIDIA A40 GPUs were used for training. The training procedure with both subsets (to obtain Control Set 1 and Control Set 2) was similar.

Appendix A2

*Additional quantitative metrics*

Additional to the main metric, Structural Similarity Index Measure (SSIM), four other metrics are tested to check robustness of the metrics. Similarity metrics can be roughly divided into three types: pixel similarity, structural similarity, and perceptual similarity. Whereas pixel similarity metrics look at pixel-by-pixel absolute differences, structural similarity metrics also consider the spatial relationships. Perceptual similarity metrics use information from a deep layer of a classification deep learning network when the images pass through. Therefore, as a sensitivity analysis, we additionally estimated a metric of pixel intensity (Point Signal to Noise Ratio), another metric of structural similarity (Multiscale SSIM [7]), and two metrics of perceptual similarity (Frèchet Inception Distance [8]; and Learned Perceptual Image Patch Similarity [9]). The impact of the primary set and two Control Sets was tested for statistical significance using a one-way ANOVA followed by a post-hoc Tukey’s test.

*Additional quantitative metrics - Results*

In Table A2 the quantitative results of all metrics on the three obtained synthetic sets (primary set, Control Set1, Control Set 2) are shown. The additional similarity metrics showed a significant difference for both Control Set1 vs primary set (р < 0.001), Control Set 2 vs primary set (р < 0.001) and Control Set 1 vs Control Set 2 (p < 0.001). This indicated that this difference is driven by both subsets (Multiscale SSIM: F = 376.33, р < 0.001, Learned Perceptual Image Patch Similarity: F = 518.60, р < 0.01, and Point Signal to Noise Ratio: F = 2703.70, р < 0.001).

**Supplemental References**

1. Lloyd S. Least squares quantization in PCM. IEEE Trans Inf Theory. 1982;28(2):129–137. doi: 10.1109/TIT.1982.1056489.

2. Lorensen WE, Cline HE. Marching cubes: A high resolution 3D surface construction algorithm. Proc 14th Annu Conf Comput Graph Interact Tech - SIGGRAPH ’87. New York, New York, USA: ACM Press; 1987. p. 163–169. doi: 10.1145/37401.37422.

3. Armato SG, McLennan G, Bidaut L, et al. The Lung Image Database Consortium (LIDC) and Image Database Resource Initiative (IDRI): A Completed Reference Database of Lung Nodules on CT Scans. Med Phys. 2011;38(2):915–931. doi: 10.1118/1.3528204.

4. Park T, Liu M-Y, Wang T-C, Zhu J-Y. Semantic Image Synthesis With Spatially-Adaptive Normalization. 2019 IEEE/CVF Conf Comput Vis Pattern Recognit. IEEE; 2019. p. 2332–2341. doi: 10.1109/CVPR.2019.00244.

5. Kingma DP, Ba J. Adam: A Method for Stochastic Optimization. 3rd Int Conf Learn Represent ICLR 2015 - Conf Track Proc. 2014;1–15.

6. Heusel M, Ramsauer H, Unterthiner T, Nessler B, Hochreiter S. GANs Trained by a Two Time-Scale Update Rule Converge to a Local Nash Equilibrium. Adv Neural Inf Process Syst. 2017;6629–6640.

7. Wang Z, Simoncelli EP, Bovik AC. Multiscale structural similarity for image quality assessment. Thrity-Seventh Asilomar Conf Signals, Syst Comput 2003. 2003. p. 1398–1402. doi: 10.1109/ACSSC.2003.1292216.

8. Heusel M, Ramsauer H, Unterthiner T, Nessler B, Hochreiter S. GANs Trained by a Two Time-Scale Update Rule Converge to a Local Nash Equilibrium. Adv Neural Inf Process Syst. 2017;6629–6640.

9. Zhang R, Isola P, Efros AA, Shechtman E, Wang O. The Unreasonable Effectiveness of Deep Features as a Perceptual Metric. Proc IEEE Conf Comput Vis Pattern Recognit. 2018;(1):13.

Supplemental Tables

Table A1 - Information about number of scans, from which scanner, number of images and number of 2D images (containing a nodule) for the three sets of train data.

|  | Primary Set | Control Set 1 (2%) | Control Set 2 (0.3%) | Test set |
| --- | --- | --- | --- | --- |
| Number of scans: | 805 | 25 | 1 | 90 |
| - GE Medical Systems | 607 | 25 | 1 | 61 |
| - SIEMENS | 132 | 0 | 0 | 16 |
| - Philips | 60 | 0 | 0 | 13 |
| - TOSHIBA | 6 | 0 | 0 | 0 |
| Number of 2D images total / with nodules | 39,803 / 6,833 | 843 / 191 | 133 / 8 | 5,121 / 787 |

Table A2 – Metric results obtained using the three sets; primary, Control Set 1, and Control Set 2 (100%, 2%, 0.3% of all train data respectively). Synthetic images are assessed using Point Signal to Noise Ratio (PSNR), Structural Similarity Index Measure (SSIM), Multi-Scale SSIM (MS-SSIM), Frèchet Inception Distance (FID) and Learned Perceptual Image Patch Similarity (LPIPS). The arrow indicates if higher or lower values correspond to a better result.

|  | PSNR ↑ | SSIM ↑ | MS-SSIM ↑ | FID ↓ | LPIPS ↓ |
| --- | --- | --- | --- | --- | --- |
| 100% of Train data | 25.90 ± 2.26 | 0.78 ± 0.04 | 0.86 ± 0.04 | 9.89 | 0.20 ± 0.05 |
| 2% of Train data | 24.44 ± 1.98 | 0.78 ± 0.05 | 0.85 ± 0.04 | 23.53 | 0.22 ± 0.04 |
| 0.3% of Train data | 22.07 ± 2.33 | 0.76 ± 0.06 | 0.83 ± 0.05 | 58.23 | 0.24 ± 0.04 |

Supplemental Figures


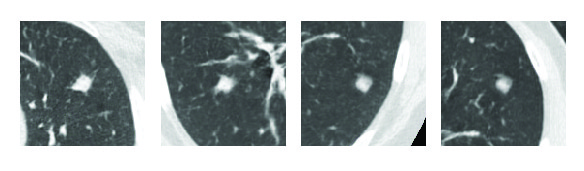


Figure A1: Four examples of a nodule cropped from synthetic images. Although the location of the nodule varies, the nodule keeps a similar appearance in texture.
